# Genetic Variability of the European Corn Borer, *Ostrinia nubilalis,* Suggests Gene Flow Between Populations in the Midwestern United States

**DOI:** 10.1673/031.008.7201

**Published:** 2008-11-14

**Authors:** Jeffrey T. Krumm, Thomas E. Hunt, Steven R. Skoda, Gary L. Hein, Donald J. Lee, Pete L. Clark, John E. Foster

**Affiliations:** ^1^ Syngenta Crop Protection, 100 JC Robinson Blvd, Waterloo, NE 68069; ^2^ University of Nebraska Department of Entomology, Haskell Agricultural Laboratory, 57905 866 Road, Concord, NE, 68728, USA; ^3^ USDA-ARS-SPA Screworm Research Unit, Panama City, Republic of Panamá; ^4^ University of Nebraska Department of Entomology, Panhandle Research and Extension Center, 4502 Ave 1, Scottsbluff, NE, 69361, USA; ^5^ University of Nebraska Department of Agronomy, Lincoln, NE, 68583 - 0915, USA; ^6^ Monsanto Company, 800 North Lindbergh Blvd., St. Louis, MO, 63167, USA; ^7^ University of Nebraska, Department of Entomology, Insect Genetics Laboratory, 312 F Plant Industry Building, Lincoln, NE, 68583 - 0816, USA

**Keywords:** molecular genetics, polymorphism, AFLP

## Abstract

The European corn borer, *Ostrinia nubilalis* (Hübner) (Lepidoptera: Crambidae), is a widely distributed and serious economic pest to corn production in the U.S. Genetic variability of *O. nubilalis* was studied in 18 sub-populations in the upper Midwestern United States using amplified fragment length polymorphism. The relatively low G*ST* values indicate that more variation exists within populations than between populations. High gene flow *(Nm)* values were indicated across the entire *O. nubilalis* population; the lowest degree of gene flow was in the northern samples (Nm = 1.96) and the highest degree of gene flow was in the southern samples (Nm = 2.77). The differences observed in the respective regions (north vs. south) may be explained by the voltinism patterns (univoltine vs. multivoltine, respectively) of *O. nubilalis:* southern multivoltine populations have opportunities for multiple matings for the duration of the year, further mix alleles. AMOVA results also indicated that most of the genetic variation was within sub-populations (≈ 81% of total variation); less variation (≈ 13%) was detected among populations within each of the three regions as designated for this study. However, the most striking and unexpected result was the low percentage of variation between all groups (≈ 6%), further supporting implications of a high degree of gene flow. These results provide support for current requirements of refugia corn planting in Bt-corn management. These results also indicate that if resistance to Bt were to evolve in *O. nubilalis,* quick action would be necessary to deter the rapid spread of the gene for resistance.

## Introduction

The European corn borer, *Ostrinia nubilalis* (Hübner) (Lepidoptera: Crambidae), was introduced into the United States, and is a widely distributed pest that has proven to be a major biotic constraint for maize development and production. *O. nubilalis* is known to be polyphagous, attacking many herbaceous plants with stems large enough for the larvae to enter. Lewis (1975) reported 223 plant species (both monocotyledon and dicotyledon) on which the borers can develop. The existence of *O. nubilalis* in the United States was first reported by Vinal (1917), however, *O. nubilalis* is thought to have been introduced multiple times to North America in shipments of broom corn from Italy and Hungary into the eastern United States and Canada between 1909–1914 (Caffrey and Worthley 1927). Since its introduction, it has become one of the most destructive insect pests of maize in North America.

*O. nubilalis* exhibits considerable genetic diversity. For example, voltinism differences between populations in North America were recognized shortly after the insect was discovered. Voltinism associated with diapause is an inherited characteristic modified by environmental factors. The wide geographic distribution exposes *O. nubilalis* to ecological conditions that differ in photoperiod, temperature, host plant availability, and growing season length (Calvin et al. 1991). Populations can be characterized as univoltine, bivoltine, and multivoltine (Showers et al. 1989). Ikten (2002) found voltinism to be genetic in origin, sex-linked, and controlled by a few loci. Further, Ikten (2002) found voltinism displayed a response to short photoperiods whereby *O. nubilalis* could adapt quickly to local conditions. As a result, historically bivoltine populations can become univoltine by a simple drop in temperature during critical days of diapause giving the species the flexibility to take advantage of the full growing season depending on altitude and latitude.

While pesticides have helped control insect pests of food and fiber, pesticide resistance will occur for any pest control product with high selection pressure that removes susceptible individuals from the population and allows only those individuals that possess resistance genes to reproduce with no other intervention (Clark and Yamaguchi 2002). Biotechnological developments, such as the isolation of genes with insecticidal properties and the ability to insert those genes into sexually incompatible species, have allowed scientists and producers to diminish insect damage to plants. An effective application of biotechnology has been the expression of bacterial insecticidal toxins from *Bacillus thuringiensis* Berliner (Bt) in several plant species (i.e. maize, cotton) where they can provide control of lepidopteran insect pests such as *O. nubilalis.* With current demands by producers for Bt crops, there is potential for the development of resistance to plant mediated Bt toxins. It is important to develop better understanding of the insect's genetic structure, genetic variation, and gene flow that can provide the basis for improvement and changes in current management strategies for insect control and resistance management.

A great deal of research has been devoted to the biology and behavior of *O. nubilalis,* driven by the need to determine the affects of *O. nubilalis* injury to crops, particularly corn, *Zea mays,* to assess economic damage and to develop management strategies (Hunt et al. 2001). However, limited information is available about *O. nubilalis* genetic variation, gene diversity, and gene flow. Previous studies comparing allozymes (Harrrison and Vawter 1977; Carde et al. 1978; Cianchi et al, 1980; and Glover et al. 1990) of pheromone and voltinism races found limited genetic variation while Marcon et al. (1999) using PCR-RFLP found similarity among *O. nubilalis* populations. From a resistance management standpoint, this has major implications for selection pressure in localized areas resulting in a higher likelihood of resistance to pesticides including plant-mediated Bt. However, Pornkulwat et al. (1998) and Saldanha (2000) found evidence of genetic variability among *O. nubilalis* populations using random amplified polymorphic DNA (RAPD) markers. Bourguet et al. (2000) also studied gene flow of French populations of *O. nubilalis* and found a high and homogenous gene flow. More recently, Coates et al. (2004) found significant genetic differentiation between Atlantic coast and Midwestern United States samples. But Bazin et al. (2006) indicated that there does not appear to be a detectable correlation between mtDNA polymorphism and species abundance; mtDNA may reflect the time of the last selective sweep rather than the demographics or population history of the organism and, concluded that mtDNA diversity is unpredictable making it not useful for biodiversity studies.

Genetic variation, governed by natural selection through the interaction of genetic forces and changing environments through space and time, provides the basis for evolutionary change. Genetic differentiation between populations largely depends on the interacting balance between gene flow, genetic drift, and natural selection (Futuyma and Peterson 1985). Although natural selection acts directly on phenotype, it is a major factor causing genetic differentiation at the protein and DNA level. Studies of genetic variability used to infer gene flow could help determine if the entire *O. nubilalis* population is truly a single interbreeding population in which intense gene flow can occur.

The AFLP technique has proven to be a reliable tool to generate highly polymorphic molecular markers used to study genetic divergence in insect populations such as: *Spodoptera frugiperda* (McMichael and Prowell, 1999; Suinaga et al. 2004; Clark *et al.* 2006), *Lymantria dispar* (Reineke et al. 1999), and *Bemisia tabaci* (Cervera et al. 2000). The AFLP technique combines the reliability of RFLPs and power of PCR in a single technique. Garcia et al. (2004) compared RAPD, RFLP, AFLP, and SSR markers and concluded that AFLP markers were the best choice for evaluating the diversity and genetic relationships between tropical maize lines without requiring previous knowledge of any DNA sequencing. Alamalakala (2002) found that AFLP consistently distinguished between European and North American populations of *O. nubilalis* providinga good starting point for further insect genetic studies using AFLPs for *O. nubilalis.*

The objective of this study was to measure genetic variation within and between sub-populations to infer genetic diversity and gene flow for *O. nubilalis.* Current information on gene flow is limited, but knowledge of the degree of genetic variation and gene flow of *O. nubilalis* is imperative to develop effective management strategies for crop protection.

**Table 1.**
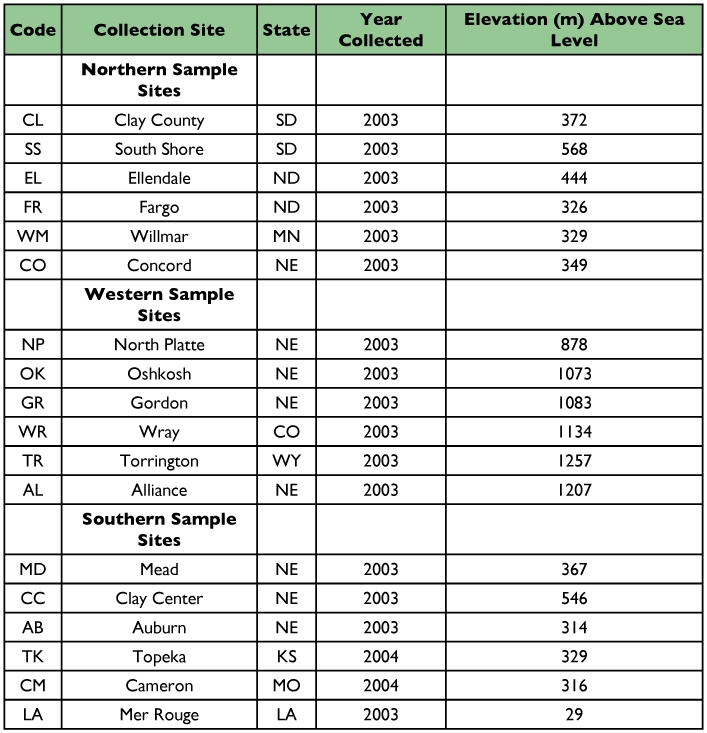
Code, collection site, and year of collection of *O. nubilalis* populations.

## Materials and Methods

### Sample collection

**Figure 1.**
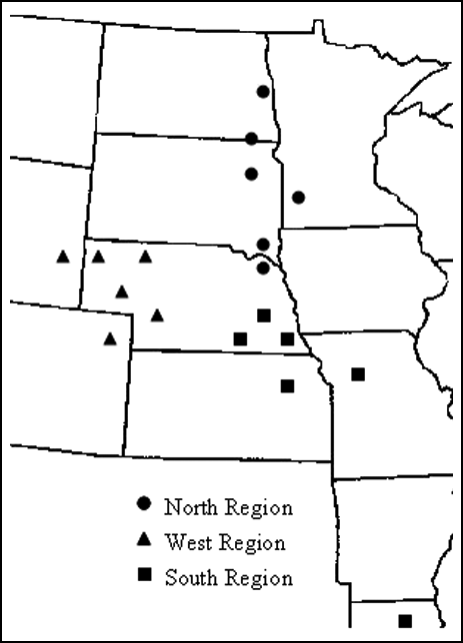
Sample distribution of *O. nubilalis* sub-populations across the upper Midwestern United States and in Louisiana.

Twenty specimens were collected from 17 sub-populations (locations) in eight states in the upper Midwest and one location from Louisiana (LA) (Table 1; Figure 1). Specimens were frozen in an ultra-low freezer to kill them quickly, maintain DNA integrity, and for long term storage. All samples, except from Louisiana, were over-wintering 5th instars collected in the fall from maize which allowed all ecotypes (univoltine, bivoltine, multivoltine) of *O. nubilalis* to be present. The LA adult samples were collected from rice in mid summer.

### DNA isolation and quantification

DNA was isolated from 10 of the 20 individually frozen insect samples using a modified Black and Duteau (1997) CTAB extraction protocol. Prior to homogenization, the gut was removed from the larvae; and the head, wings, and abdomen were removed from adult insects from Mer Rouge, LA. The procedure was modified with the addition of a chloroform/phenol extraction step prior to ethanol precipitation. After removal of ethanol, the pelleted DNA was dissolved in 50 *µ*l 1X TE buffer (10mM Tris-HCL pH 8.0; 0.1 mM EDTA).

Quantification to a known concentration of *λ* DNA (Life Technologies, www.invitrogen.com) was performed by loading 1.0 **µ**l of DNA mixed with 1.0 **µ**l tracking dye to a 1% agarose gel that was electrophoresed for 30 min. Ethidium bromide stained gels were then visualized using Advanced Quantifier version 4.0 (www.genomicsolutions.com). DNA samples were diluted with TE buffer if concentrations were >22.5 ng//** µ**l.

### AFLP-PCR

The AFLP procedure was achieved using 3 steps: 1) DNA template preparation; 2) DNA template preamplification and; 3) AFLP Selective amplification. Template preparation and AFLP assays were performed using a modified protocol of Vos et al. (1995). Infrared labeled (IRD-700) (LI-COR, Lincoln, NE, USA) *Eco*RI primers were used in the polymerase chain reaction.

### Template preparation

Approximately 160 ng of genomic DNA in 7 ***µ***l of volume was incubated with restriction endonucleases *Eco*RI and *Mse*I (New England Biolabs, www.neb.com) for 2.5 hrs at 37°C in a total volume of 12.5 ***µ***l which contained 1.25 ***µ***l of 10X One-Phor-All buffer (Amersham Pharmacia biotech, www.apbiotech.com), 0.125 ***µ***l of 10 U/***µ***l*Mse*I enzyme (1.25U/reaction), 0.0625 ***µ***l of 20 U/***µ***l*Eco*RI enzyme (1.25 U/reaction), 0.125 ***µ***l of 10 mg/ml BSA (bovine serum album) (New England Biolabs), and autoclaved nanopure water to make up the volume to 12.5 ***µ***l. The restriction fragments were then incubated for 11.5 hrs at ∼ 25°C with 5 ***µ***l ligation mixture containing 0.15 ***µ***l of T4 DNA ligase, 0.5 ***µ***l of 10X T4 ligase buffer (New England Biolabs), 0.5 ***µ***l of 5 pmoles/ ***µ***l*Eco*RI adapter, 0.5 ***µ***l of 5 pmoles/***µ***l MseI adapter (Operon Technologies, www.operon.com), and 3.35 ***µ***l autoclaved nanopure water. After ligation, the template was diluted 8-fold. Double stranded adapters for ligation to the corresponding cut made by the restriction enzyme were prepared by incubating equimolar amounts of both adapter strands for 10 min at 65°C, 10 min at 37°C, 10 min at 25°C and stored at -20°C.

### Preamplification of DNA template

PCR amplification of the ligated material consisted of 20 cycles (30s at 94°C, 1 min at 56°C, and 1 min 72°C) using 1.0 ***µ***l of diluted template from adapted DNA diluted 8-fold with IX TE buffer (10mM Tris-Cl, 0.1 mM EDTA [pH8.0]), 8 ***µ***l pre-amp primer mix II (contained two oligonucleotide primers, one corresponding to the *Eco*RI adapted ends and one corresponding to the *Mse*I ends; Life technologies, www.operon.com), 1.0 ***µ*** l of 10X PCR buffer containing 15mM MgCl_2_ and 0.25 ***µ***l of 5 *U*/***µ***l Ampli*Taq* DNA polymerase (1.25 U/reaction) (Applied Biosystems, www.appliedbiosystems.com). The oligonucleotide primers in the pre-amp primer mix II are complementary to the adapter/restriction site with *Mse*I primer containing one selective nucleotide M(N+1) primer and *Eco*RI primer containing no selective nucleotide E(N+O) primer (Table 2). Products from the preamplification reactions were diluted 20-fold with autoclaved nanopure water and were used as the template for selective amplification reactions.

### Selective PCR amplifications

After screening 24 primer combinations, five primer combinations (Table 3) were selected for AFLP analysis. Reaction volumes of 10.5 ***µ***l containing 2.0 ***µ***l of diluted preamplified template, ∼ 5.0 ***µ***l of autoclaved nanopure water, 1.2 ***µ***l of 10X PCR buffer containing 15 mM MgCl2, and 0.06 ***µ***l of 5 U/ ***µ***l AmpliTaq polymerase (Applied Biosystems), 2.0 ***µ***l of MseI primer (M+1+2) (6.7 ng/ ***µ***l dNTPs) (Life Technologies), and 0.2–0.5 ***µ***l of 1.0 pmoles/ ***µ***l IRD-700 labeled EcoRI primer (LI-COR, www.licor.com). Selective amplifications were conducted in a DNA thermal cycler 9600 (Applied Biosystems) using the following Touchdown PCR program: 1 cycle of 30s at 94° C, 30s at 65° C and 1 min at 72° C; 12 cycles of 30s at 94° C, 30s at 65°C, (subsequently lowering the annealing temperature by 0.7°C per cycle) and 1 min at 72° C; 23 cycles of 30s at 94° C, 30s at 56° C, 1 min at 72° C. After selective amplifications, reactions were stopped by adding 2.5 ***µ***l blue stop solution (LI-COR).

**Table 2.**
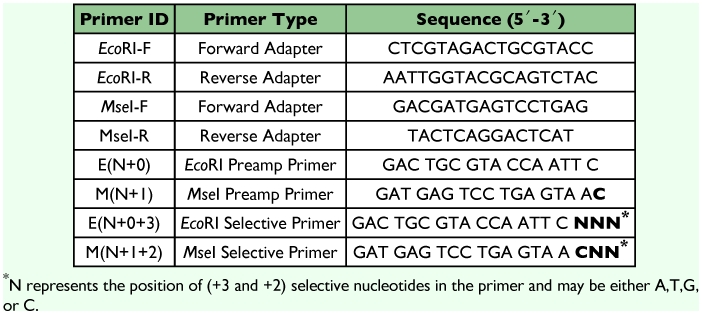
Oligonucleotide adapters and primers used for AFLP analysis.

**Table 3.**
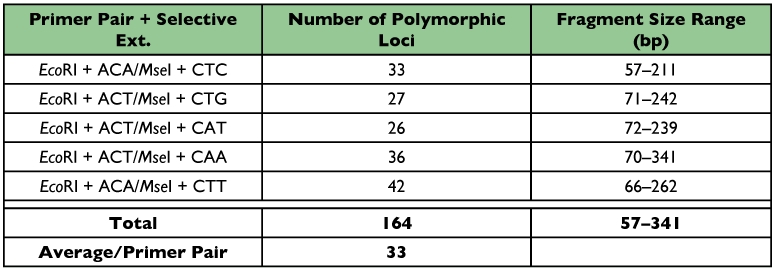
Selective Primers used for AFLP analysis their associated amplified loci and range of fragment sizes for *O*. *nubilalis.*

Samples were then denatured at 95°C for 3 min and flash cooled on ice immediately before loading the polyacrylamide gel for electrophoresis. One ***µ***l of the sample was electrophoresed through KBplus 6.5% ready-to-use gel matrix (LI-COR), and the infrared fluorescent bands were then detected by a laser scanning system LI-COR Model 4200S-2.

### Scoring AFLP data

Sizes of AFLP fragments were estimated using an IRD-700 labeled 50–700bp ladder. Fragments were scored using SAGA MX version 3.2 (LI-COR); a software program designed specifically for scoring AFLP data. The data were then converted to numerical data (1s for band presence, 0s for band absence) that allowed the identification of polymorphic markers. There were five gels for each primer pair containing four populations randomly picked for a total of 25 PAGE gels. All gels were viewed for clarity prior to scoring to establish scorable bands at a given fragment size for all populations. All bands consistently recognized by SAGA Generation 2 Software Version 3.2 (LI-COR), both monomorphic and polymorphic, were scored and included in the binary data matrix.

### Coefficient of variation analysis

Analysis was conducted using DBOOT version 1.1 (Coelho 2001) to assess the appropriate number of polymorphic loci required for acceptable precision for genetic analyses. Bootstrapping analysis using the simple matching coefficient (Kosman and Leonard 2005) was conducted with 1000 permutations to assess the strength of the molecular markers. A comparison was then made between the coefficient of variation values against the number of markers scored and was then plotted.

### Genetic diversity and gene flow of *O*. *nubilalis*

Data were analyzed using POPGENE version 1.32 (Yeh and Boyle 1997) using a dominant marker data set (164 markers) assuming Hardy-Weinberg equilibrium. Samples were grouped into three geographical regions due to proximity. Analyses were observed at three levels (1) individuals; (2) regions; (3) and whole populations. The percent (%) polymorphism, genetic diversity or heterozygosity (H), GST, and gene flow estimation (Nm) were then assessed within and between all populations. Individual populations were analyzed for genetic diversity (H) for each sub-population as per Nei (1973). GST values generated by POPGENE were given for the whole population and were expressed as the gene diversity of the single population subtracted from the gene diversity of the total population divided by the gene diversity of the total population (GST ^=^ HT - HS / HT). Gene flow was estimated from GST values and is expressed as (N*m*) = 0.5(1 - GST)/GST (McDermott and McDonald 1993).

### Mantel tests comparing genetic dissimilarity and geographical distance

A permutation test (Mantel 1967) was conducted to test the relationship between geographical distance and the measure of genetic dissimilarity (1-similarity), to further verify the estimated levels of gene flow found in the POPGENE analysis. The Mantel permutation tests were conducted using the MxCOMP module in the NTSYSpc ver. 2.1 (Rohlf 2002) software program with 1000 permutations. Tests were first conducted on the full data set and then separately with western samples, northern samples, and the southern samples as data sets.

### Analysis of molecular variance

Analysis of molecular variance (AMOVA; Excoffier et al. 1992) was conducted with ARLEQUIN 2.0 (Schneider et al. 2000). In this analysis, total variance of the AFLP data set was partitioned at three hierarchical levels: (1) an among-population component; (2) a regional or six sub-population component; (3) and a within-population component. Unlike the calculations used for Nei's GST values, the AMOVA partitions the variation according to correlations among genotypes rather than gene frequencies due to the dominant expression of AFLP markers. The significance of the three variance components was tested using 1000 random permutations. A two-part AMOVA analysis was conducted to test genetic divergence (FST) as a factor of variation among individuals within a given population and between populations.

#### Principal component analysis (PCOA)

Principal component analysis was used to test genetic isolation between sampled populations using NTSYSpc ver. 2.1 (Rohlf 2002). This two-dimensional analysis, an Eigen plot, helps identify patterns in the data and enables one to graph the results highlighting the similarities and differences.

#### UPGMA cluster analysis

An Unweighted Pair Group Method with Arithmetic Mean (UPGMA) consensus cluster analysis as outlined by Sneath & Skoal (1973) was conducted using NTSYSpc ver. 2.1 (Rohlf 2002) on all 18 sub-populations of *O. nubilalis* to illustrate genetic similarity. Bootstrap analysis, using BOOD-P software ver. 3.1 (Coelho 2001), was used to test the reliability of the dataset with 10,000 pseudoreplicates.

### Results

#### Number and size of AFLP loci observed

The five primer combinations used with DNA from 18 sub-populations and 180 individual samples of *O. nubilalis* produced 164 amplified loci, averaged 33 loci per primer combination, and fragment size ranged from 57–341 bp (Table 3). Polymorphism within the 18 sub-populations was high, averaging 83% (136 out of 164 were polymorphic) and ranging from 72% to 94% (Table 4), indicating that AFLP provides polymorphic markers for studying genetic variation.

**Table 4.**
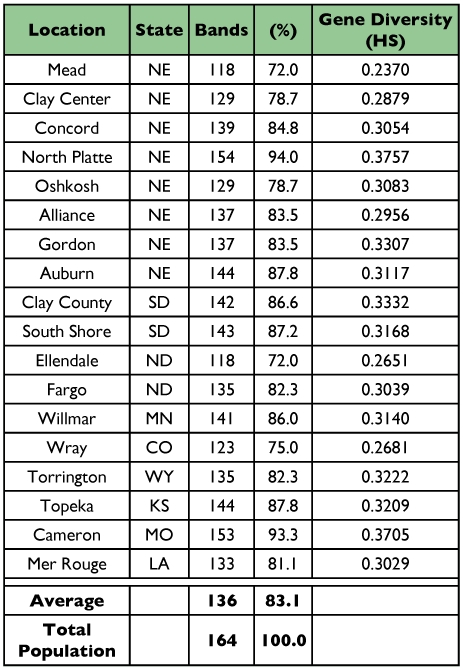
Heterozygosity and polymorphism (%) for 18 subpopulations of *O*. *nubilalis.* Individual heterozygosity (HS) indicates a lack of homogeneity.

#### Coefficient of variation of AFLP loci (markers) explored

**Figure 2.**
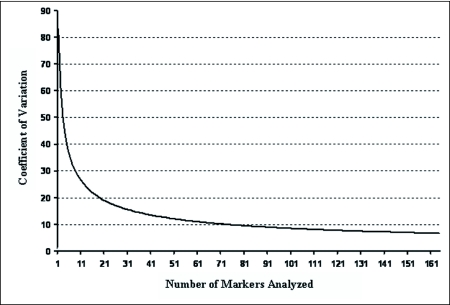
The coefficient of variation of *O. nubilalis* populations using AFLP molecular markers with 1000 bootstrap replications. Greater than 90% of the variation in the *O*. *nubilalis* population is explained by these AFLP markers.

The correlation of the coefficient of variation and the number of molecular markers examined for the sampled population is important for determining robustness in genetic variability studies because either a large number of loci or a large number of individuals should be examined (Hoelzel 1995). Analysis using the DBOOT program showed that the high number of markers used decreased the coefficient of variation to the point that all but 6.7% of the variation in the population was explained (Figure 2). Therefore, 164 markers were sufficient for unbiased genetic analyses.

#### Genetic diversity, GST values, and gene flow

Genetic diversity, or population heterozygosity (H), values calculated were high from all sub-populations, ranging from 0.2370 at Mead, (NE) to 0.3757 at North Platte, (NE); overall gene diversity averaged 0.3094 (Table 4). Analysis across all populations revealed a high genetic diversity value of 0.4121 (Table 5). The GST value among all sampled populations was low, 0.1652, indicating a high degree of within population variation (83%) and low variation among populations (16.5%). Nm values were high in all three regions (Table 5), suggesting that events, such as migration, provide opportunity for interbreeding and gene flow.

#### Permutation tests comparing genetic dissimilarity and geographical distance

Results indicated no evidence of correlation between genetic distance and geographical distance (data not shown). There was no correlation for the western samples (r = -0.3765, t = 1.484, P = 0.9312), northern samples (r = 0.2937, t = 1.1087, P = 0.8662), southern samples (r = 0.2546, t = 0.9671, P = 0.8332), nor for the full data set combined (r = -0.0146, t = -0.1223, P = 0.4513). These results further imply there is not genetic isolation in this portion of *O. nubilalis* range and agree with results of Coates et al. (2004).

#### Analysis of molecular variance (AMOVA)

The AMOVA analysis showed that approximately 81% of the variation in the data set was from genotypic variation within populations (Table 6). Only 13% of the variation could be attributed to differences among populations within regions while the remaining 6% was due to the variation among regions. The genetic divergence (FST) value for 18 sub-populations was 0.1907 indicating low differentiation between populations. These results were concordant to those obtained using POPGENE (Table 5). Similar results were reported by Coates et al. (2004); this low level of population differentiation supports the possibility of gene flow across this portion of the *O. nubilalis* range.

#### Principal component analysis (PCOA)

**Figure 3.**
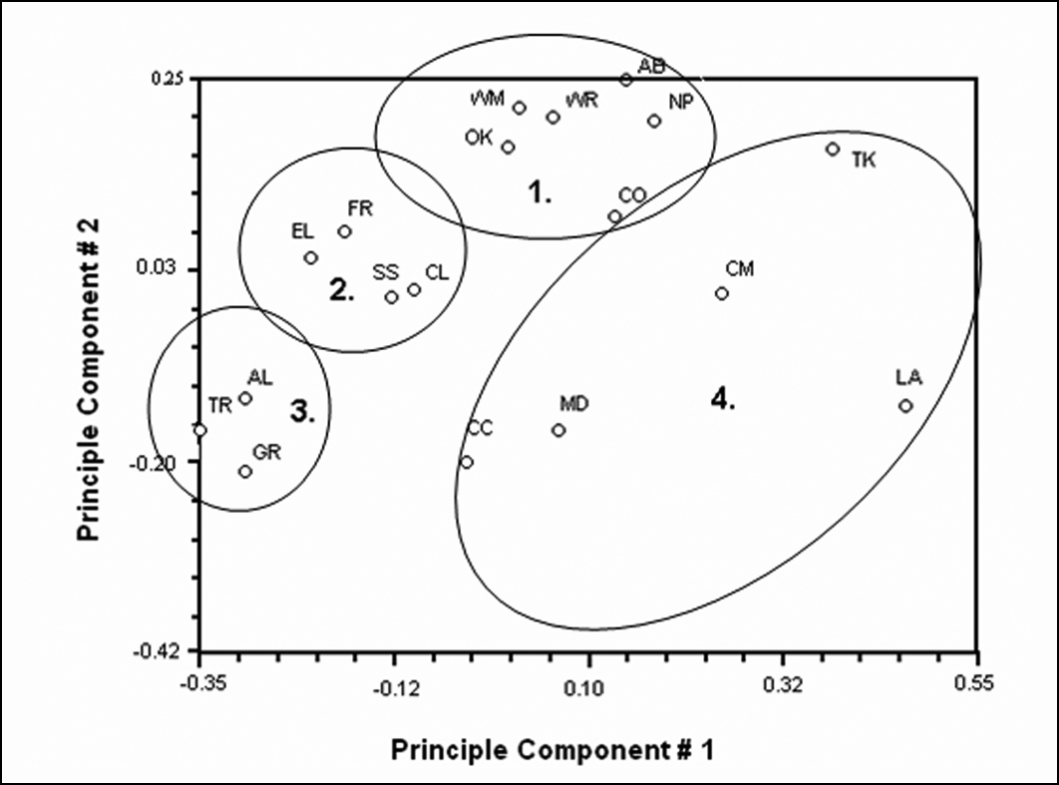
Principal Component Analysis (PCOA) of 18 sub-populations of *O. nubilalis.* Four distinct clusters were evident possibly indicating some genetic isolation from other clusters.

Principal component analysis for these 18 sub-populations of *O. nubilalis* revealed four distinct clusters (Figure 3). Cluster 1 consisted of mixed populations from several states that may represent areas of transition from univoltine to bivoltine ecotypes, cluster 2 represented univoltine samples from South Dakota and North Dakota, cluster 3 represented samples typical of the univoltine ecotype, and cluster 4 represented samples that were less similar (more scattered on the graph) that are southern populations probably representing the transition from bivoltine to multivoltine ecotype.

**Table 5.**
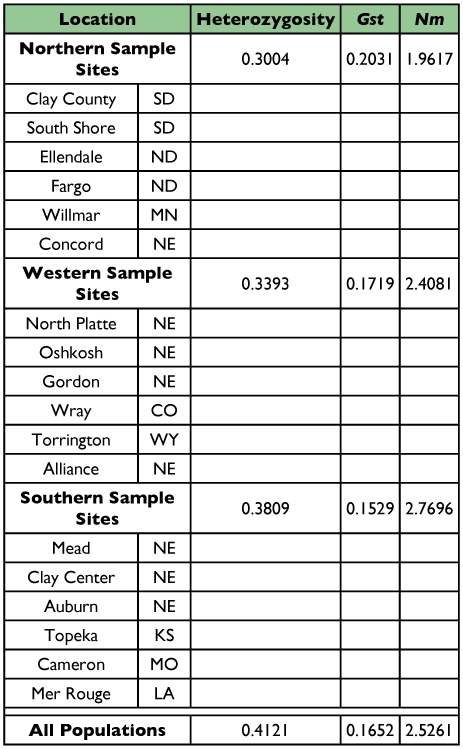
Pair wise comparisons of Nei's coefficient of gene differentiation (G*ST*) between three regions of *O*. *nubilalis* populations and estimates of gene flow (N*m*).

#### UPGMA cluster analysis

**Figure 4.**
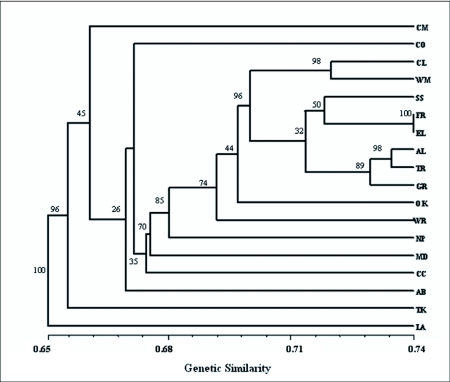
UPGMA Dendrogram of 18 sub-populations of *O. nubilalis.* Bootstrap values are at each node.

Consensus averages yielded a dendrogram with similarity coefficients ranging from 65% to 74% for all 18 sub-populations of *O. nubilalis,* indicating a high degree of variability within locations (Figure 4). Bootstrap values ranged from 26 – 100% but a majority of nodes had bootstrap values exceeding 70%. Most sub-populations clustered with others in close geographic proximity or with similar climate. For example, samples from the western region (higher altitude) and from the northern region (higher latitude), with a similar level of growing degree days and suspected to consist primarily of a univoltine ecotype, were more genetically similar than samples from southern locations. Those nodes showing lower bootstrap values (AB, CC, CM and CO) indicate weak association and could represent areas of transition with a mixture of ecotypes (particularly bivoltine and multivoltine) as is also indicated with the PCOA analysis (Figure 3). As reported by Pornkulwat et al. (1998), univoltine ecotypes, using current techniques, may be more easily identified than bivoltine or multivoltine ecotypes, indicating that gene flow may be somewhat restricted for the univoltine ecotype.

**Table 6.**
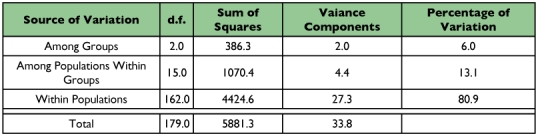
Hierarchical analysis of molecular variance (AMOVA) for 18 sub-populations of *O*. *nubilalis.* The majority of the variation is within a given population.

### Discussion

Molecular genetic tools have proven successful at detecting genetic variability. Pornkulwat et al. (1998) used RAPD markers that were able to distinguish multivoltine from univoltine and bivoltine ecotypes. Saldanha (2000) used RAPD-PCR to distinguish between local populations of *O. nubilalis*and found a large genetic group consisting of univoltine, bivoltine, and multivoltine ecotypes in Nebraska. However, disadvantages of RAPDs are apparent and therefore results can be arguable. Alamalakala (2002) showed that using AFLPs allows the analysis of large numbers of loci while repeatability is greatly increased over RAPDs. Our AFLP studies evaluated numerous loci showing a very high degree of polymorphism between the 18 sub-populations of *O. nubilalis* studied.

Understanding population structure of *O. nubilalis* will provide critical base-line information for developing sustainable management strategies. Populations may either exist as a single panmictic unit, as if in Hardy-Weinberg equilibrium, where all individuals have the equal opportunity to randomly mate, they may occur as a series of small sub-populations which are isolated from one another (island model), or they may exist as a continuous population where genetic exchange takes place by geographically proximate individuals and there is some genetic isolation by distance (Slaktin and Barton, 1989). Frankham et al. (2002) indicated that a GST value of 0.15 indicates differentiation among fragments. At the population level, our study indicated a GST value of 0.1652. Therefore, *O. nubilalis* in the Midwest may not consist of a continuous population as some differentiation may be taking place, as discussed by Coates et al. (2004), but this is far removed from representing an island model. Studies by Bourguet et al. (2000) also concluded there is a high degree of gene flow and no differentiation between northern (univoltine) and southern (bivoltine) populations in France: observed and expected heterozygosities were almost identical 0.19 to 0.33 and 0.23 to 0.32 respectively based on six polymorphic allozyme markers.

Like the GST analysis, AMOVA results indicated that a majority of the variation detected was within sub-populations. Only a small amount of variation was due to variation among the three regions studied (north, south, and west). However, the most striking and unexpected result was the low percentage of variation among groups, further enhancing the implication of a high degree of gene flow possibly through migration events. The degree of variation within or among populations was variable. For many species, variability may be greater between than within populations (Roderick 1996; Clark *et al.* 2006). Local selection pressures and barriers could provide for genetic isolation resulting in a high degree of genetic drift. However, previous studies showed a higher degree of variation within populations than between populations for many insects (Coates and Hellmich 2003, Juan et al. 2004, Timmermans et al. 2005). Results from our study examining *O. nubilalis* populations from the Midwestern U.S. using AFLPs show a larger degree of genetic variation than previously reported but is similar to results found by Bourguet et al. (2000) with *O. nubilalis* in Europe.

The numerical differences observed in Nm between respective regions (north vs. south) and respective UPGMA clustering may be explained by the voltinism patterns that prevail in those regions. The opportunity for multiple mating during the year in southern regions could result in high genetic variability. Another possible explanation of lower indications of gene flow in the northern region could be that *O. nubilalis* living on alternate hosts, such as *Solanum* and *Amaranthus* may be unable to mate with individuals from maize due to different emergence patterns of *O. nubilalis* from the alternate hosts compared to those from maize. Or, as suggested by Coats et al. (2004), there may be genetic differences between sympatric uni- and bivoltine ecotypes. Coats et al. (2004) evaluated individuals using a portion of mtDNA whereas we examined 164 loci from random locations across the genome of 180 individuals. It would be beneficial to conduct similar studies over a broader range using a greater number of individuals for genetic analyses using AFLP that provides the polymorphic markers necessary to better address the questions of genetic differentiation in *O. nubilalis.*

Showers et al. (2001) conducted mark and recapture studies to track movement of *O. nubilalis* after release. They found 37–52% of *O. nubilalis* males recaptured flew 800 m or more, 8–11% flew 3.2 km or more, while some collections of marked males and females occurred at distances greater than 40 km. However, a very large percentage of the *O. nubilalis* released were never recaptured (over 99.6%). It is possible that some of these individuals traveled much further than placement of the recapture pheromone traps. Showers et al. (1995) reported similar findings while studying potential movement on surface airflow of *O. nubilalis.* Although indirect measures of gene flow are tenuous (Whitlock and McCauley 1998, Hey and Machado 2003) our results, along with previous results (Showers et al. 1995, 2001) illustrating that *O. nubilalis* were capable of dispersing at least 32 km/yr, would support the concept of gene flow within a continuous population. Results from these ecological and genetic studies provide support for current refuge requirements relative to the use and management of Bt-corn. Also, these results indicate that if insect resistance was to evolve in *O. nubilalis,* quick action will need to be taken to defer the spread over large geographic areas.

## Abbreviations

**AFLP**: amplified fragment length polymorphism
**RAPD**: random amplified polymorphic DNA
